# “PICS”: a novel patient-specific landmark for thalamic surgical interventions in the posterior limb of the internal capsule signal

**DOI:** 10.3389/fnhum.2025.1707031

**Published:** 2025-12-02

**Authors:** Rémi Patriat, Jayashree Chandrasekaran, Karianne Sretavan, Henry Braun, Samuel Brenny, Yasamin Seddighi, Joshua E. Aman, Meghan Hill, Jerrold L. Vitek, Noam Harel, Leonardo Almeida

**Affiliations:** 1 Center for Magnetic Resonance Research, Department of Radiology, University of Minnesota, Minneapolis, MN, United States; 2 Department of Biomedical Engineering, University of Minnesota, Minneapolis, MN, United States; 3 Graduate Program in Neuroscience, University of Minnesota, Minneapolis, MN, United States; 4 Department of Neurology, University of Minnesota, Minneapolis, MN, United States; 5 Department of Neurosurgery, University of Minnesota, Minneapolis, MN, United States

**Keywords:** DBS, DiMANI, direct targeting, patient-specific, diffusion MRI, internal capsule

## Abstract

**Introduction:**

Difficulties in direct visualization of thalamic subnuclei are likely a contributor to inconsistent surgical outcomes among patients with medication refractory tremors. We present a new MRI landmark, represented by a bright signal in the posterior limb of the internal capsule signal (PICS), that can serve as a consistent marker for indirect location of the Vim nucleus of the thalamus. We evaluated the visibility of PICS across multiple MRI sequences at 7Tesla (T) and 3T, and its anatomical characteristics were identified using tractography.

**Methods:**

One healthy control and 15 essential tremor (ET) patients were scanned. To characterize the PICS fibers, two posterior limb of internal capsule (pLIC) tractography schemes were conducted with cortical ROIs as seeds and the pLIC as a waypoint: (i) gross motor cortical ROIs, (ii) M1 and S1 homunculus. Finally, intra- and post-operative clinical data were merged for one ET DBS patient to show correspondence between the parcellation results and clinical observations.

**Results:**

PICS was consistently identified across multiple MRI sequences. Tractography analyses identified PICS to correlate with the distribution of motor fibers from the internal capsule. For the M1 homunculus, two somatotopic clusters were observed: one including mostly trunk, lower and upper limbs; and another, more anteriorly, with head/face clustering with tongue/larynx. For the S1 homunculus, the trunk region was overall the most posterior region followed by the upper limb/face anteriorly and Area2. Intra-operative stimulation at two different depths resulted in pLIC-specific side effect in the tongue/face. At those depths, measurements showed closer proximity of the DBS electrode to M1 clusters of head/face and tongue/larynx, validating the imaging findings.

**Conclusion:**

PICS appears to be a reliable radiological marker comprising cortico-spinal tracts, in isolation from corticobulbar tracts fibers. It is consistently located lateral to the Vim, making it a potential landmark to infer Vim location and help refine targeting for thalamic procedures. The parcellations of the pLIC using M1 homunculus could potentially inform lead or ablation location based on side effect profiles (e.g., head/face/tongue vs. trunk/limbs). Therefore, proximity or distance to PICS may potentially guide lead placement to avoid procedure-related capsular side effects while optimizing benefits.

## 1 Introduction

Essential tremor (ET) is the most prevalent movement disorder, impacting individuals at all ages, including approximately 4% of adults over the age of 65 (Louis, 2005). ET can greatly impact the quality of life with tremors affecting not only the upper limbs, but also the lower extremities, trunk, head and speech (Zesiewicz et al., 2010; Whaley et al., 2007). Unfortunately, up to 50% of ET patients either become refractory to or unable to tolerate medications at the doses required to achieve adequate symptom control. For refractory cases, more advanced treatments including lesional therapies and neuromodulation such as gamma knife, high- and low-intensity focused ultrasound (FUS), or deep brain stimulation (DBS) can be considered (Zesiewicz et al., 2011; Koller and Vetere-Overfield, 1989).

The most common neuromodulation option offered to ET patients is DBS. In this procedure, an electrode is implanted in the region of the ventrointermediate subnucleus of the thalamus (Vim), and electrical current is continuously delivered through selected contacts of the DBS lead. Also, in recent years, thalamotomies targeting the Vim using magnetic resonance-guided focused ultrasound (MRgFUS) have received increased interest because it is a less invasive FDA-approved procedure (Ferreira Felloni Borges et al., 2023). Although these interventions can be very effective, standard DBS and lesioning approaches often yield variable outcomes with tremor improvements ranging from 35 to 80% (Giordano et al., 2020), sometimes leading to repeat surgeries (Agrawal et al., 2021; Rolston et al., 2016; Ferreira Felloni Borges et al., 2023). Some groups have also reported a gradual loss of therapeutic efficacy over time (so-called habituation), which can start as early as 6 months post-surgery (Fasano and Helmich, 2019; Ferreira Felloni Borges et al., 2023). Reports of habituation vary widely across ET DBS studies, ranging from no habituation (Cury et al., 2017) up to affecting 73% of Vim-DBS cases over time (Shih et al., 2013). Lastly, non-invasive approaches such as lesional therapies preclude intraoperative mapping (e.g., microelectrode recording (MER)), as well as testing of the implanted electrode (as done with DBS), which are opportunities of refinement of the surgical targeting to ensure more consistent outcomes.

**Figure 1:**
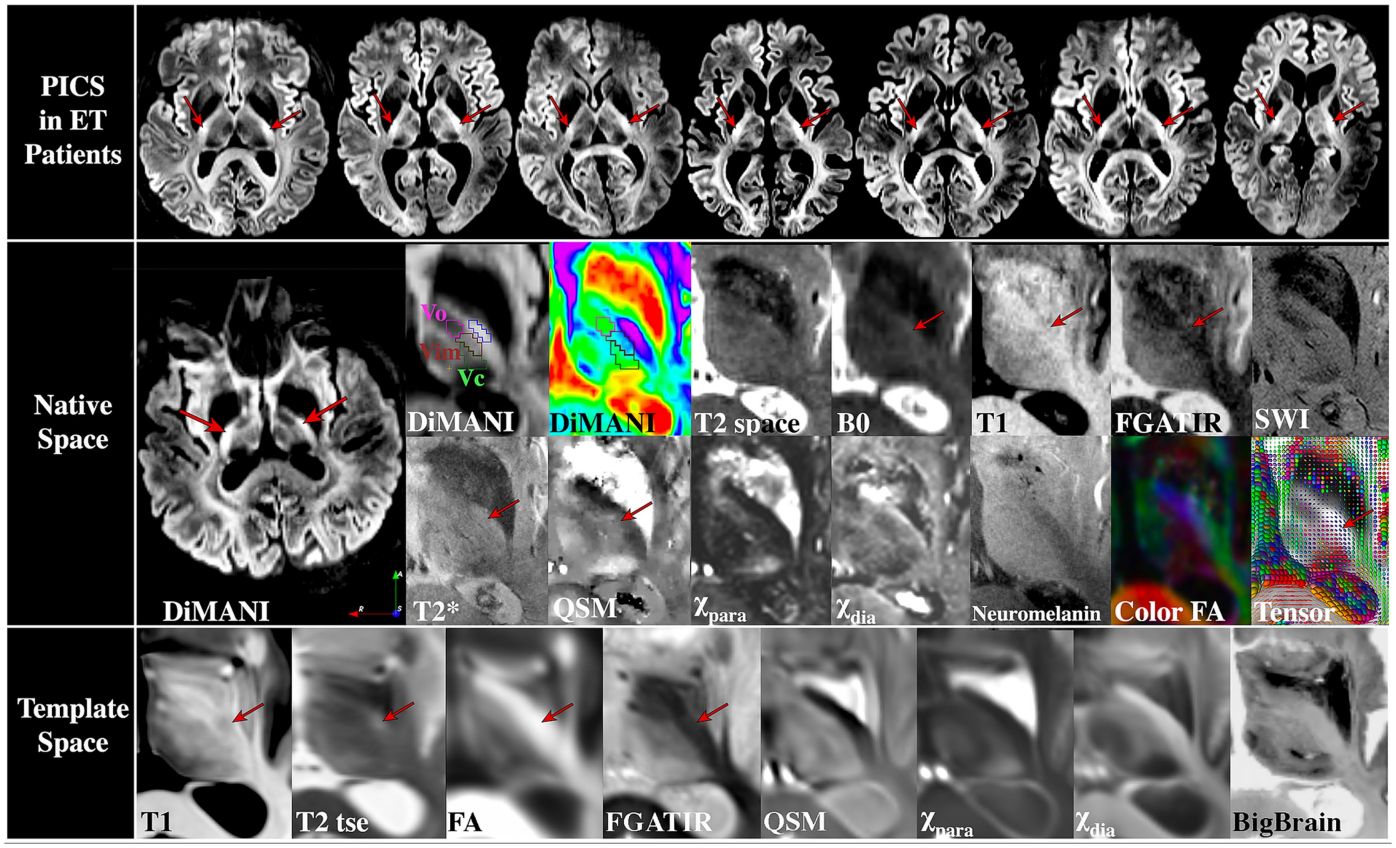
Visibility of PICS (top) on DiMANI images across ET patients, (middle) in a variety of MR contrasts in one healthy participant, and (bottom) on templates. Red arrows indicate where PICS is visible. Vop = posterior ventralis oralis nucleus, Vim = ventrointermediate nucleus, Vc = ventral caudalis nucleus. Outlines on DiMANI images: Pink = Vop, Red = Vim, Green = Vc, Blue = PICS.

Some of the variability in clinical efficacy may arise from challenges in directly visualizing and targeting specific thalamic subnuclei because of the inter-individual variability in the size, shape, and geometric configuration of these subnuclei. Several imaging methods have been proposed to directly visualize thalamic subnuclei including susceptibility-weighted imaging (SWI) (Abosch et al., 2010; Najdenovska et al., 2019), quantitative magnetic susceptibility mapping (QSM) (Chiang et al., 2018), white-matter nulled T1 imaging, such as FGATIR (Sudhyadhom et al., 2009), 3D-EDGE (Middlebrooks et al., 2021), and WMn-MPRAGE (Su et al., 2019), as well as a new diffusion MRI (dMRI) method called DiMANI (Patriat et al., 2024). Each one of these methods has its limitations, ranging from clinical feasibility (acquisition, processing and expertise availability) to lack of sufficient independent clinical validation. Patriat et al. (2024), demonstrated that, by using the DiMANI method, it is possible to directly visualize the subnuclei of the thalamus, potentially facilitating surgical targeting of these structures by using the “zebra pattern” (Patriat et al., 2024). Further, a hyperintense posterior limb of the internal capsule signal (PICS), was consistently visualized on DiMANI images (Figure 1), warranting further investigation as a potential radiological landmark to improve surgical targeting accuracy.

The present study aims to evaluate the visibility of PICS across multiple MRI sequences, and identify its anatomical characteristics using tractography in order to determine its potential to serve as a clinical and radiological landmark to refine Vim targeting in lesional or neuromodulatory procedures.

## 2 Methods

### 2.1 Subjects

Fifteen ET patients were enrolled from the University of Minnesota DBS program. Inclusion criteria required patients to have a diagnosis of ET and be suitable candidates for Vim-DBS surgery, as defined by the institution’s standard of care practice. The study did not interfere or change the patients’ routine treatment protocol. These participants completed a research-only 7 Tesla (7 T) MRI scan prior to surgery. Additionally, we obtained 3 T data from one of our patients. However, our institution clinical workup only includes a 3-direction DTI dataset which is insufficient for computing a meaningful DiMANI image; therefore, we downloaded 3 T diffusion data from the Human Connectome Project (HCP – Subject 100,610) to show that PICS is visible using DiMANI at 3T. The participants’ demographics are summarized in Table 1. Additionally, one healthy volunteer (M, 72 years old) was recruited to enable collection of MR images that are not typically included in our clinical 7 T protocol. The study was approved by the Institutional Review Board at the University of Minnesota and informed consent was obtained from all participants prior to inclusion in the study. All experiments were performed in accordance with relevant guidelines and regulations.

**Table 1:** Demographics information and pLIC volumes.

Study subject	Sex	Age at 7T	Disease duration (yrs)	Left pLIC Volume (mm^3^)	Right pLIC Volume (mm^3^)
ET001	M	70	49	4,694	5,133
ET002	F	66	5	3,946	4,034
ET003	M	65	1	3,561	4,338
ET004	M	41	17	5,293	5,170
ET005	M	73	5	4,679	4,934
ET006	M	67	26	4,762	4,492
ET007	F	73	30	4,072	4,286
ET008	M	55	21	4,519	5,042
ET009	F	73	2	5,215	5,109
ET010	F	64	33–43	5,584	4,833
ET011	M	70	4	4,914	5,654
ET012	M	72	20	5,002	5,205
ET013	F	63	4	3,546	3,811
ET014	M	42	4	5,480	5,168
ET015	F	72	23–33	3,539	3,317

### 2.2 MRI acquisition and processing

All participants were scanned at the Center for Magnetic Resonance Research on a 7 T Siemens Magnetom Terra MRI scanner using SC72 gradients capable of 70 mT/m and a 200 T/m/s slew rate, driven by a Siemens console (Erlangen, Germany). The images were acquired with a 32-element head array coil (Nova Medical, Inc., Burlington, MA). The MRI clinical protocol for ET patients included: Diffusion-weighted images, T1 MPRAGE, FGATIR, and SWI. Additionally, to assess whether PICS was visible on other MRI contrasts, we also acquired the following scans for the healthy volunteer: QSM, T2*, Neuromelanin, and multi-echo MP2RAGE in order to generate a synthetic WMn-MPRAGE. Table 2 lists the MRI protocol parameters. The 3 T dataset was acquired on a Siemens Prisma system using gradients capable of 80 mT/m and a 200 T/m/s slew rate, driven by a Siemens console (Erlangen, Germany). Scans included: T1, T2 dark fluid, T2tse, T2space and T2blade.

**Table 2:** MRI scanning parameters.

Sequence	Resolution (mm)	TR (ms)	TE (ms)	TA	Acceleration	Coverage	Comments	Subjects
Diffusion	1.25 × 1.25 × 1.25	6,500	71	2 × 6min 30 s	GRAPPA = 2, Multi-band = 2	Whole Brain	50 directions, b-value = 1,500 s/mm2, 5 additional b0-volumes, 2 acquisitions with opposite phase encoding direction	ET Patients, Healthy Volunteer
MPRAGE	0.6 × 0.6 × 0.6	3,100	3.48	5 min 08 s	GRAPPA = 3	Whole Brain		ET Patients, Healthy Volunteer
FGATIR	0.8 × 0.8 × 0.8	3,000	2	8 min 43 s	GRAPPA = 2	Whole Brain	TI = 430 ms	ET Patients, Healthy Volunteer
SWI	0.4 × 0.4 × 0.8	210	14	7 min 33 s	GRAPPA = 2	Axial Slab (48 slices)	In-plane interpolation to 0.2 × 0.2 × 0.8	ET Patients, Healthy Volunteer
QSM	0.6 × 0.6 × 0.6	26	3.6/7.2/10.8/14.4/18/21.6	6 min 36 s	GRAPPA = 4	Whole Brain		Healthy Volunteer
T2*	0.4 × 0.4 × 1	1,150	20	5 min 13 s	GRAPPA = 2	Axial slab (42 slices)		Healthy Volunteer
Neuromelanin	0.4 × 0.4 × 1.5	400	2.27	8 min 46 s		Whole Brain	2 averages	Healthy Volunteer
me-MP2RAGE	0.7 × 0.7 × 0.7	5,380	1.74/3.58/5.42/7.26	7 min07	GRAPPA = 4	Whole Brain	TI = 734/2890 ms	Healthy Volunteer
3T scanning parameters								
T1	1.0 × 1.0 × 1.0	2,600	3.02	4 min 50 s	GRAPPA = 2	Whole Brain		ET Patient
T2 Dark Fluid	0.4 × 0.4 × 3	9,000	94	4 min32 s	GRAPPA = 2	Whole Brain		ET Patient
T2 space	0.8 × 0.8 × 0.8	3,200	383	11 min58 s	GRAPPA = 2	Whole Brain		ET Patient
T2 tse	0.5 × 0.5 × 2	6,440	78	6 min 09 s	GRAPPA = 2	Axial slab (32 slices)		ET Patient
T2 blade	0.7 × 0.7 × 3	5,500	117	3 min 20 s		Whole Brain		ET Patient
Diffusion (HCP)	1.25 × 1.25 × 1.25	5,500	89.5	2 × 9min 41 s	Multi-band = 3	Whole Brain	90 directions, b-value = 1000,2000,3000 s/mm2, 6 additional b0-volumes, 2 acquisitions with opposite phase encoding direction	Healthy Volunteer

TR = repetition time, TE = echo time, TA = acquisition time, TI = inversion time.

dMRI preprocessing steps included: motion, susceptibility, and eddy current distortions correction using FSL’s eddy and topup algorithms. The DiMANI images were generated by computing the mean, voxelwise, of the diffusion weighted volumes (e.g., only b > 100 volumes were kept) (Patriat et al., 2024). For visualization purposes, DiMANI images were intensity equalized using an adaptive histogram equalization^1^. For the healthy volunteer, a non-linear registration between the subject’s native space and MNI template space was obtained using the HCP pipelines in order to study whether high signal-to-noise ratio average images also display PICS. Average T1, T2, FA and FGATIR images were obtained from the Lead-DBS toolbox^2^. We also used the MuSus-100 QSM template (He et al., 2023), the *χ*-separation atlas (Min et al., 2024), and the BigBrain histological atlas (Amunts et al., 2013).

### 2.3 Posterior limb of the internal capsule segmentation

The pLIC was manually segmented on the DiMANI image using Slicer3D^3^. The lateral and medial borders were defined as the pallidum/putamen and the thalamus, respectively. The anterior border was the genu of the internal capsule. The posterior, inferior and superior extent were chosen following the definition of the IC from the ICBM-DTI-81 template (Mori et al., 2008).

### 2.4 Posterior limb of the internal capsule vs. PICS region tractography

The first exploratory experiment was to generate tracts from PICS and the regions from the pLIC immediately anterior and posterior to this landmark. The purpose was to observe whether the PICS region was discriminately connected to a particular part of the cortex while the rest of the pLIC connect to other cortical regions and decide if there was merit in performing a larger scale parcellation analysis of the pLIC. This experiment was conducted in DSI studio^4^ using the heathy volunteer data, where manual segmentations of the pLIC regions (PICS and pLIC regions anterior and posterior to PICS) were used as seeds and tracts were allowed to freely propagate ipsilaterally throughout the brain. To that effect, a rectangular exclusion mask, the length of the brain in sagittal orientation going from the top of the brain down to just above the superior cerebellar peduncles (SCP), was placed at the midline. Note that this allowed for tracts to cross hemispheres at the SCP and below. The default deterministic tracking parameters were used, and tracking was stopped once 10,000 tracts were created. The default parameters, as described by dsi studio, were as such “The anisotropy threshold was randomly selected between 0.5 and 0.7 otsu threshold. The angular threshold was randomly selected from 45 degrees to 90 degrees. The step size was set to voxel spacing.”

### 2.5 Posterior limb of the internal capsule parcellation

All parcellation experiments were carried out using FSL’s probtrackx2 (Behrens et al., 2007). Each cortical ROI was used as a seed, the ipsilateral pLIC as a waypoint and all other ROIs (all contralateral and the remaining ipsilateral ROIs) as regions of avoidance. To visualize the location of all the functional territories within the pLIC, we thresholded each parcel to 80% of its maximum connectivity value instead of flowing a winner-take-all approach. This resulted in parcels with some overlap, so we also show skeletonized maps where, at each axial slice, the center of mass of the connectivity distribution is displayed for each region. This builds a center-of-mass “skeleton” in the direction that *the majority* pLIC fibers run. For this study, we conducted three parcellation experiments. First, following Archer et al. (2018; Mori et al., 2008), we used the HMAT atlas to define M1, S1, supplemental motor area (SMA), pre-SMA, as well as ventral and dorsal premotor (PMv and PMd)(Mayka et al., 2006). Since a similar approach was used to generate an atlas of the corticospinal tract parcellation (Archer et al., 2018), we also brought the SMATT to the patient’s native space to compare how this normative parcellation compares to patient-specific parcellations at the level of pLIC. Second, we used the Brainnetome atlas to define the subdivisions of M1 corresponding to lower limbs, upper limbs, trunk, tongue/larynx and head/face (Fan et al., 2016). Finally, we used the Brainnetome atlas to define the subdivisions of S1 corresponding to upper limbs, trunk, tongue/larynx and upper limbs - head/face (Xu et al., 2016). Fiber tracking settings included FSL’s default curvature threshold of 0.2, maximum number of steps per sample of 2000, number of sample equal to 5,000, step length of 0.5 mm, subsidiary fiber volume threshold of 0.01, and termination of pathways that looped back on themselves.

### 2.6 Compiling results in a common space

In order to facilitate visualization of the parcellation results across patients, we generated a study-specific template from each patient’s DiMANI and fractional anisotropy map. This was achieved using DTI-TK, which has been shown to excel at creating templates based on diffusion data (Zhang et al., 2007). The non-linear transformations to bring each patient’s native space data into template space were then used to bring parcellation results from each patient to the study-specific common space using DTI-TK.

### 2.7 DBS data

The purpose of this experiment was to use clinical data in a case example to show a within-subject correspondence between the pLIC parcellations and clinical observations, as well as provide a window to the potential proof-of-concept clinical relevance to our results. We obtained the intraoperative and post-operative data relevant to the right Vim DBS surgery for patient ET015 (Table 1). These included surgical notes containing information from microelectrode recordings (MER) trajectories and micro-stimulation, a 4-week high resolution post-operative computed tomography image (CT; free from brain shift), as well as programming notes. To reconstruct the final location of the DBS electrodes and contacts with respect to the patient’s own anatomy, the CT was non-linearly registered to the MR images using Elastix (Klein et al., 2010). From the surgical and programming notes, we extracted the information (depth, track) regarding pLIC-related side effects observed clinically and mapped those in 3DSlicer. Finally, in order to relate the clinical observations to the patient-specific pLIC parcellation results, we used the ModelToModelDistance module^5^ within 3DSlicer.

## 3 Results

### 3.1 Posterior limb of the internal capsule and PICS

Table 1 shows the volumes of manually segmented left and right ICs for all fifteen ET patients. Volume sizes were found to be similar within patient (t-test *p*-value = 0.3) but quite variable across patients with a mean of 4,587 ± 704 mm^3^ for the left IC and 4,701 ± 635 mm^3^ for the right.

**Figure 2:**
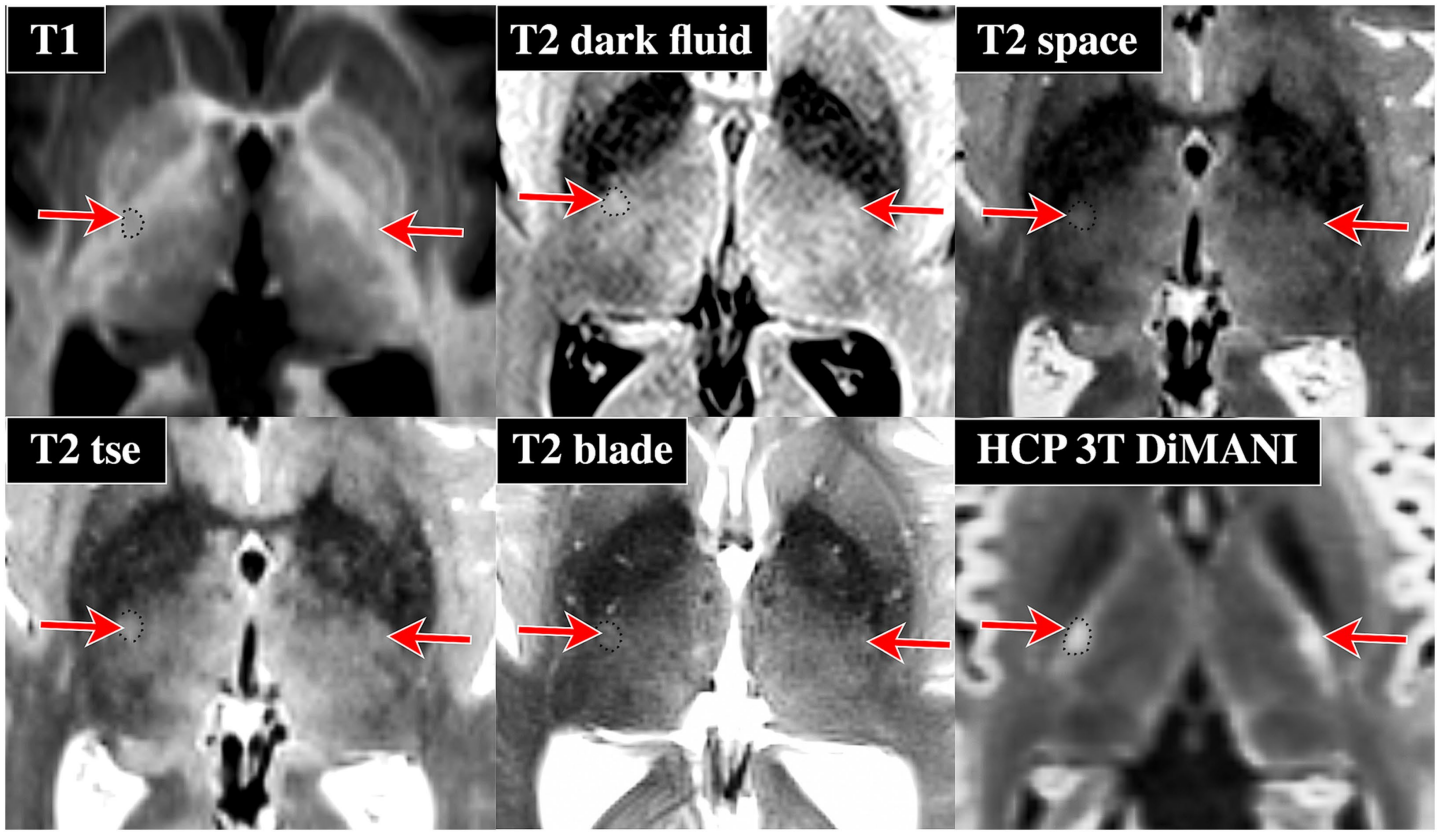
Evidence of PICS visibility on 3T data from one of the ET DBS patients. Note that we downloaded a 3 T diffusion dataset from the HCP database (subject 100,610) since our current 3 T clinical protocol does not include dMRI data, only a 3-direction DTI dataset is acquired. Red arrows indicate the location of PICS. The black dotted outline shows PICS outline on one side of the brain. Note that PICS is generally more intense than its surrounding internal capsule regions except for T1.

Figure 1 shows images from our healthy participant for a wide variety of contrasts. PICS was clearly identified on the B0 image (hyperintense) and more subtly on the T1 (MP2RAGE uni, hypointense), T2* (hyperintense), FGATIR (mildly hyperintense), Tensor, and QSM (hyperintense with respect to pLIC but not thalamus) images. However, it was not visible on the patient’s SWI, T2space, χ_para_, χ_dia_ or Neuromelanin image. PICS was clearly visible on the T1 (hypointense), T2 (hyperintense) templates, and more subtly on the FGATIR and FA templates. PICS could be easily identified in DiMANI images for all 15 patients (Figure 1). The clear visibility in some of the templates indicates that visualizing PICS might require very high signal-to-noise ratio (SNR) to be visible on some contrasts, potentially explaining its ease of localization on the DiMANI sequences. Figure 2 shows the presence of PICS at 3 T in one of our ET patients. Similarly, PICS is hyperintense in DiMANI, T2 space, T2tse, T2 dark fluid and T2blade and hyperintense in T1.

**Figure 3:**
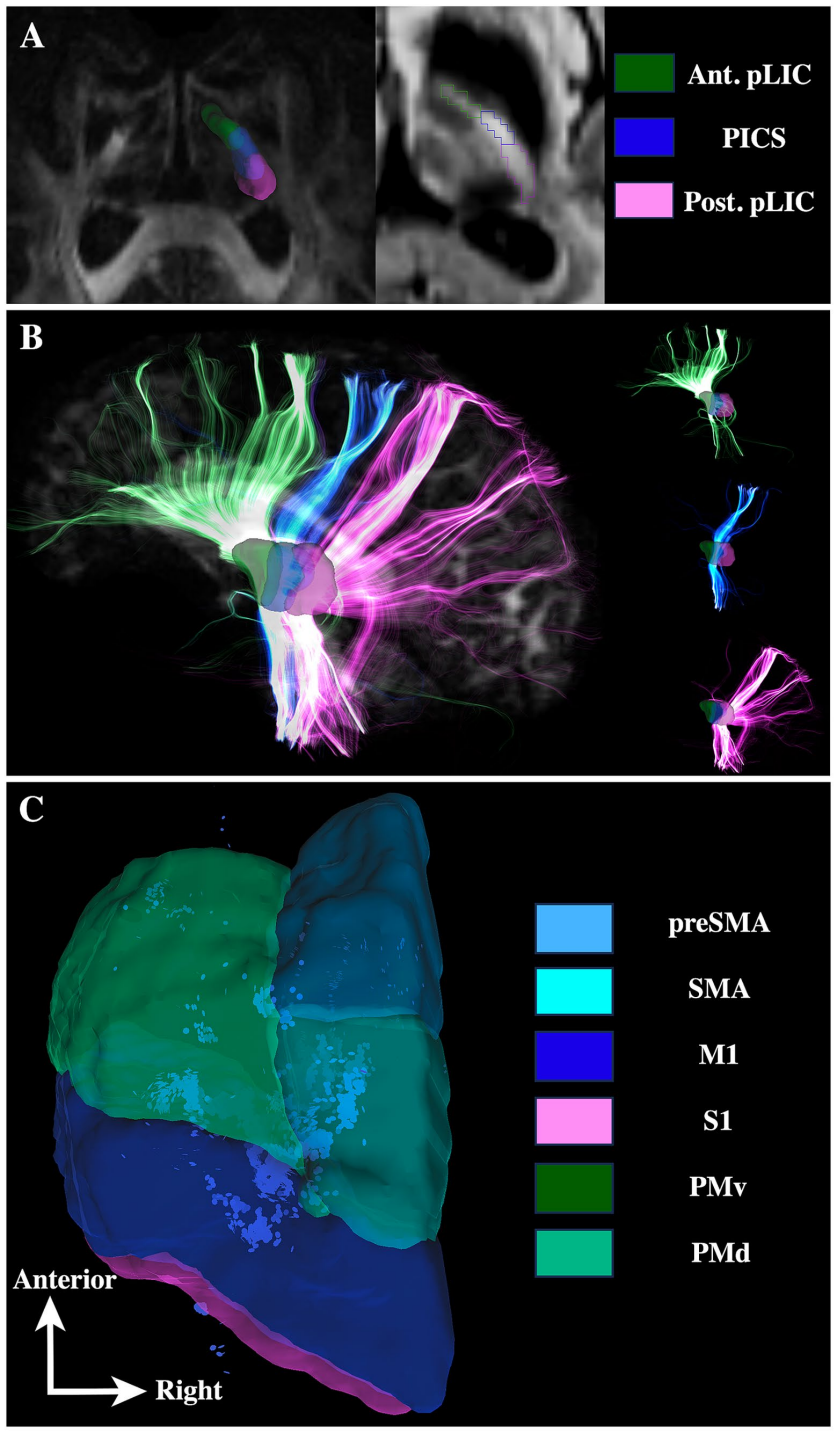
Deterministic tractography shows segregated white matter pathways for the PICS fibers with respect to anterior and posterior portions of pLIC. **(A)** Manual segmentation of the PICS, anterior and posterior regions of the pLIC. **(B)** Tracts for all these regions. **(C)** End of tract location for the PICS fibers in the cortex with respect to the HMAT atlas. Ant = anterior, Post = posterior, SMA = supplementary motor area, M1 = primary motor cortex, S1 = primary sensory cortex, PMv = ventral premotor cortex, PMd = dorsal premotor cortex.

Figure 3 shows deterministic tracts of the pLIC with respect to PICS in one participant. In this exploratory experiment, fibers passing through the pLIC reached regions all over the cortex, including frontal, precentral, postcentral and posterior areas. Subdividing the pLIC into PICS, anterior and posterior pLIC ROIs showed that the anterior–posterior organization of cortex is preserved at the level of the pLIC with motor fibers passing through PICS (Figure 3C).

### 3.2 Parcellation based on HMAT atlas

**Figure 4:**
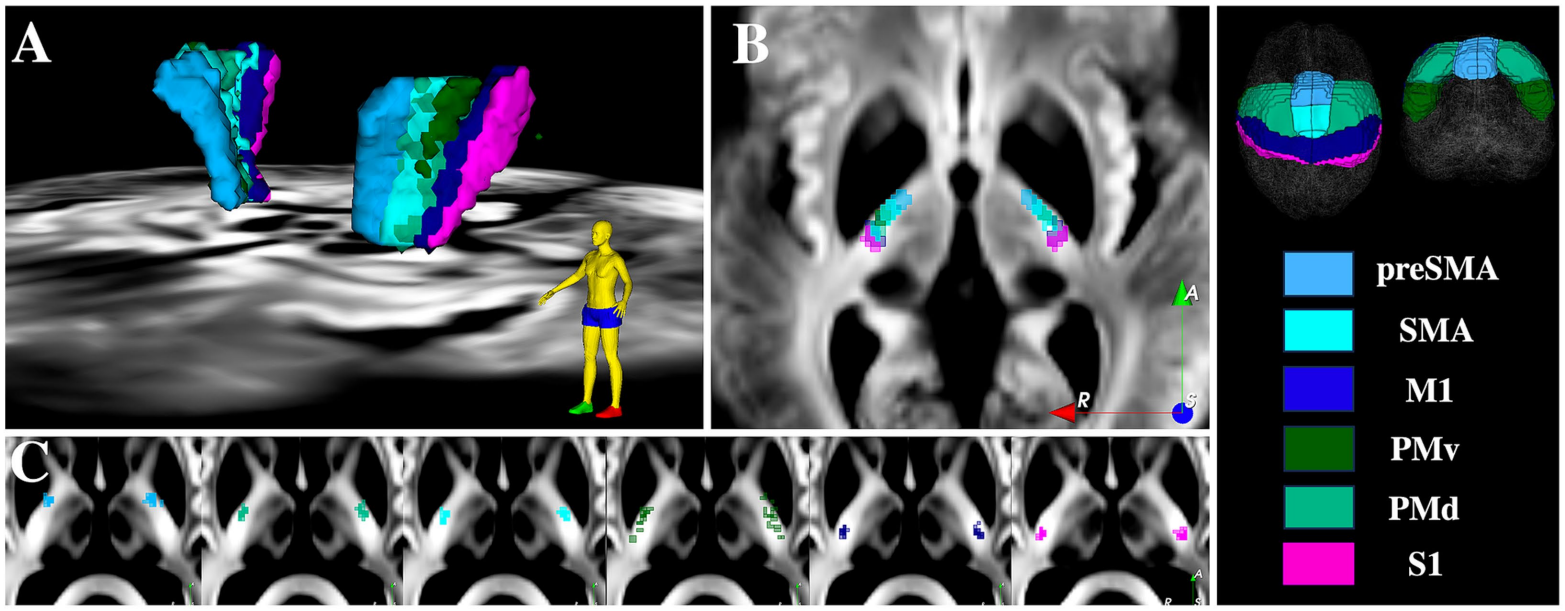
pLIC parcellation based on HMAT. **(A)** 3D visualization of the mean tractography across all 15 ET patients. **(B)** 2D visualization of the mean tractography across all 15 ET patients. **(C)** Skeletons of all individual ET patients tractography maps ordered from anteriormost to posteriormost; each dot represents one patient. SMA = supplementary motor area, M1 = primary motor cortex, S1 = primary sensory cortex, PMv = ventral premotor cortex, PMd = dorsal premotor cortex.

Figure 4 shows the pLIC parcellation results based on the HMAT definitions of M1, S1, SMA, preSMA, PMv and PMd. The average tractography results demonstrate an anterior–posterior organization in the pLIC with S1 posterior most followed anteriorly by M1, PMv, PMd, SMA and preSMA corresponding closely to the cortical organization. The regions of SMA and PMd of pLIC overlapped the most and maintained similar anterior–posterior distribution across subjects (Figure 4C). Further, the PMv region appeared to be the most variable across subjects included in our sample (Figure 4C).

**Figure 5:**
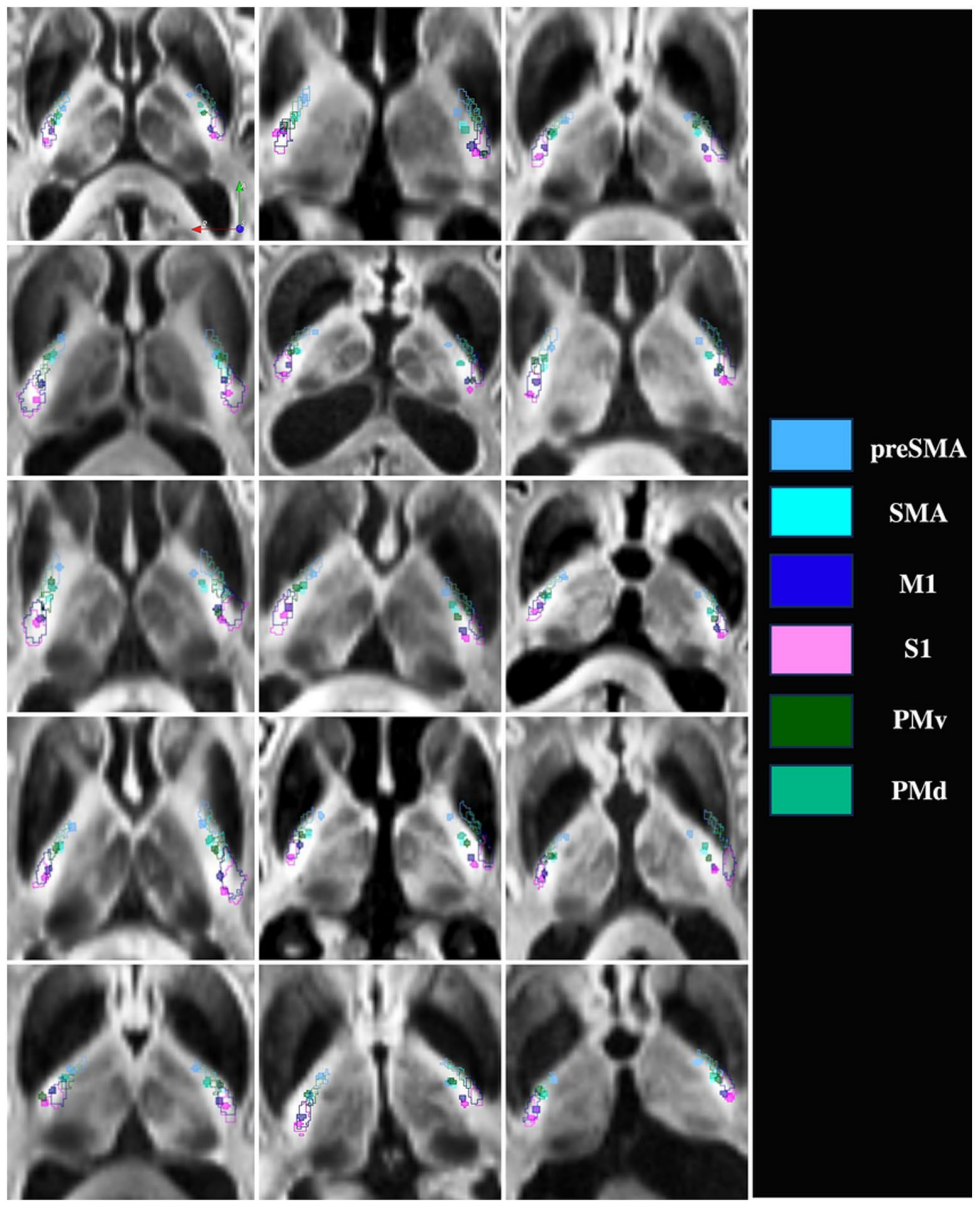
Patient-specific pLIC parcellation skeletons (filled circles) versus SMATT (outlines) for all 15 ET patients. Pink = S1, Dark blue = M1, Light blue = preSMA, Cyan = SMA, Green = PMv, Light green = PMd.

Figure 5 shows the comparison between patient-specific pLIC parcellation based on HMAT and the SMATT template registered to the patient native space. The organization and the location of the parcels in the anterior–posterior direction matched closely between the two methods. The main differences arose in the medial-lateral direction and could be attributed to potential registration inaccuracies that are typical when registering average anatomical data (e.g., MNI T1) to older, diseased, brains in which atrophy and asymmetries are commonplace, even with non-linear registration algorithms.

### 3.3 Parcellation based on M1 and S1 homunculus

**Figure 6:**
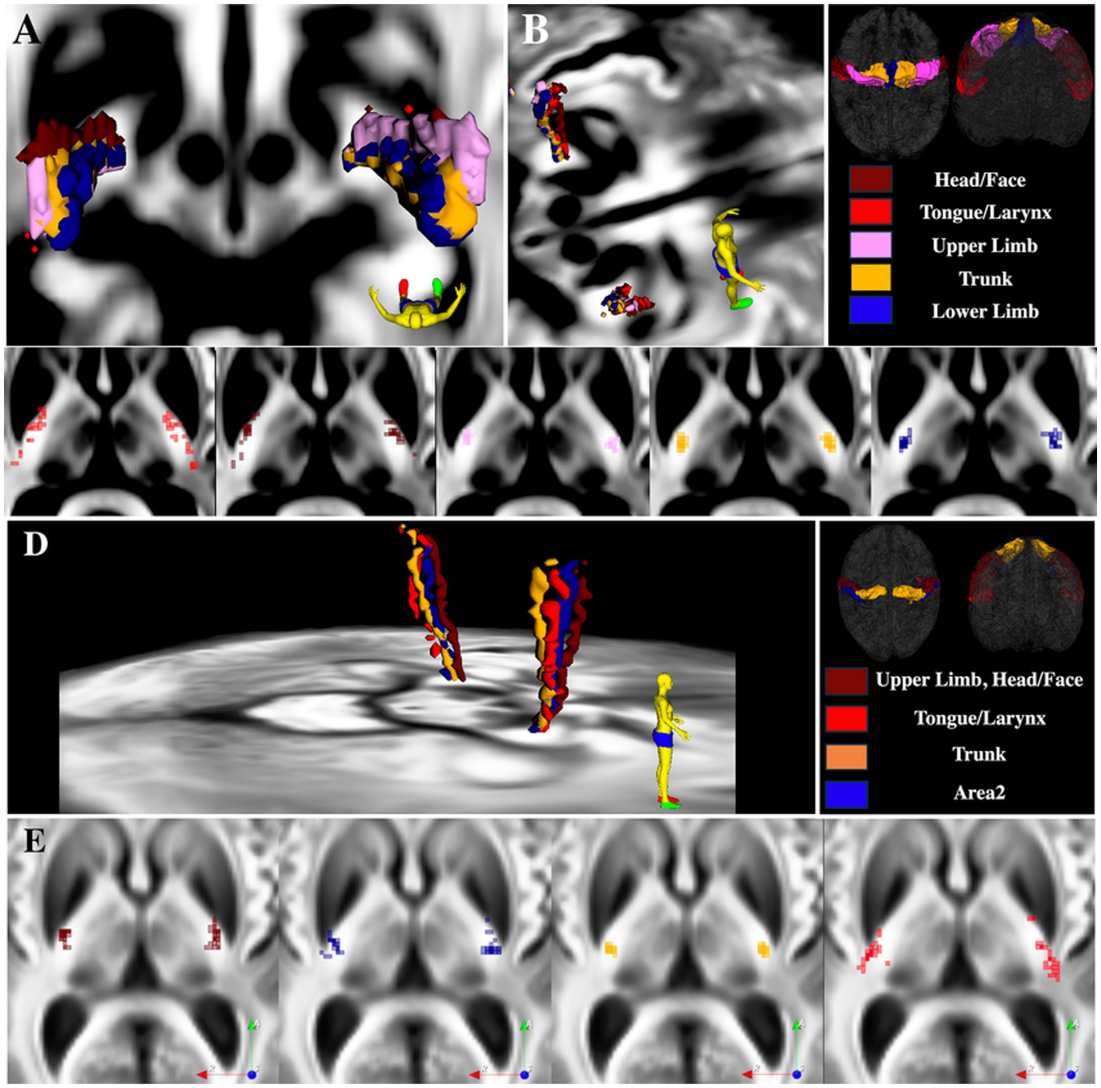
pLIC parcellation based on the M1 and S1 homunculus from the Brainnetome atlas. **(A)** 3D visualization of the mean tractography across all 15 ET patients. **(B)** 3D visualization of the mean tractography skeletons. **(C)** Skeletons of all individual ET patients tractography maps ordered from anteriormost to posteriormost; each dot represents one patient. **(D)** 3D visualization of the mean tractography skeletons. **(E)** Skeletons of all individual ET patients tractography maps ordered from anteriormost to posteriormost; each dot represents one patient.

Figures 5, 6 show the pLIC parcellation results based on the Brainnetome definitions of the homunculus of M1 and S1. Similar to the HMAT parcellation results, the S1 homunculus regions of pLIC were located posterior and lateral to M1 homunculus. As expected, overlap occurred within the homunculus regions (e.g., M1 head/face vs. M1 tongue/larynx vs. M1 trunk vs. M1 upper limb vs. M1 lower limb).

For the M1 homunculus, two somatotopic clusters were observed: one including trunk, lower and upper limbs; and another with head/face clustering with tongue/larynx. Within the trunk and limbs cluster, lower limb was found more medial to the trunk region, while upper limb was distributed more anterolateral to trunk (Figure 6). As with the HMAT parcellation experiment, the most ventral cortical ROI (tongue/larynx) was the most variable across the 15 participants. Note that the participants with M1 tongue/larynx skeletons located posteriorly typically had a focal probabilistic maps with tracts also located anterior.

For the S1 homunculus, the trunk region was overall the most posterior region followed by the upper limb/face anteriorly and Area2. The position of the tongue/larynx region of S1was the most variable across the subjects (Figure 6E).

### 3.4 Case study including data from stimulation-induced side effects

**Figure 7:**
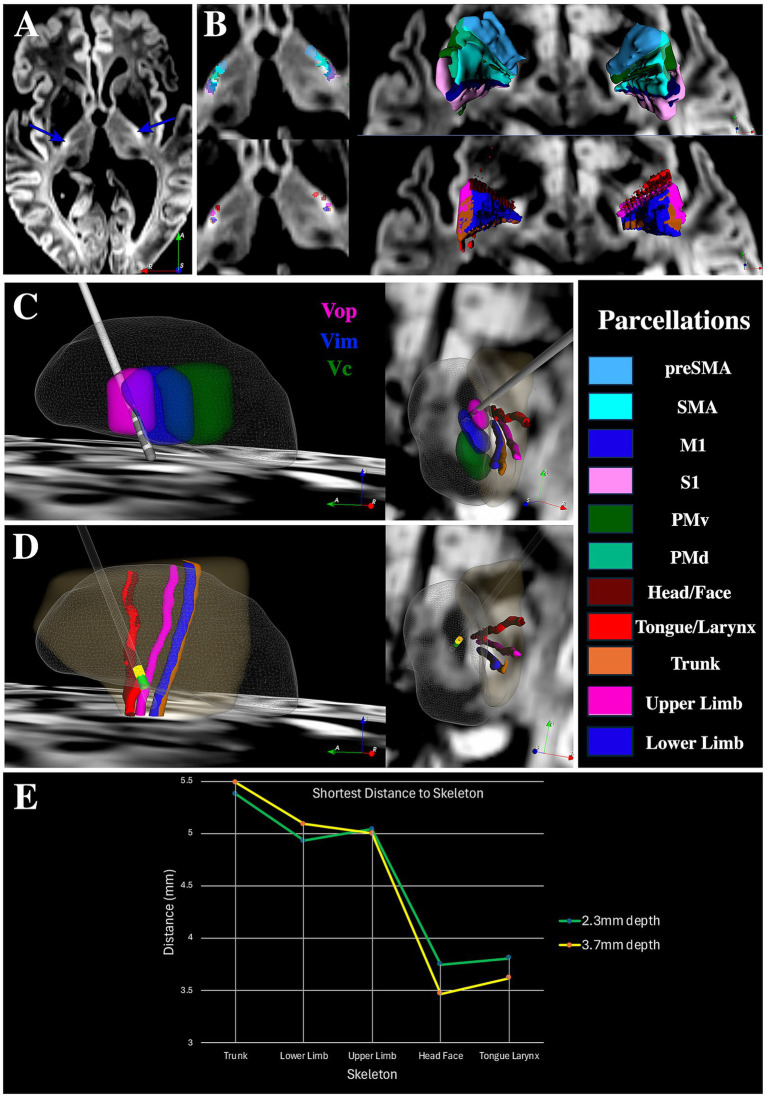
ET DBS case study. **(A)** Location of PICS on the patient’s DiMANI image. **(B)** pLIC parcellation results for this patient in an axial slice (left) and in 3D (right). **(C)** Electrode location within the thalamus (transparent mesh) with respect to Vop (pink), Vim (blue) and Vc (green) in sagittal (left) and axial (right) orientations. **(D)** Locations at which pLIC side effects were observed (yellow is 3.7 mm above target depth and green is 2.3 mm above target depth) with respect to the patient’s pLIC parcellation using the M1 homunculus (skeleton representation). The semitransparent gold shape represents the pLIC segmentation. **(E)** Shortest distance between the location where semi-microstimulation elicited pLIC side effects and the pLIC parcellation skeletons based on M1.

DiMANI images yielded an easily identifiable PICS landmark (Figure 7A). The pLIC parcellation results were similar to those described above (Figure 7B), replicating the findings from the population of 15 individuals. Final DBS electrode and contact locations corresponded to the Vop/Vim border (Figure 7C), which is typical for our center. Prior to DBS implantation, two MER tracks separated by 2 mm in the anterior–posterior direction (center and posterior BenGun holes) were recorded intra-operatively. Stimulation in the center track using the semi-micro (ring) electrode (length = 1.0 mm) located 3 mm superior to the microelectrode tip, at 3.7 mm above our target depth (130 Hz, 90 μs and 4.0 mA) and 2.3 mm above target (130 Hz, 90 μs and 4.5 mA) resulted in focal muscle contractions observable in the tongue/face, which were indicative of stimulation of the pLIC. For Vim, our target depth is at the level of the midpoint of the AC/PC line, typically equating to approximately the ventral border of the thalamus. Figure 7D shows the locations of these stimulations with respect to the patient-specific M1 homunculus parcellation of pLIC. Figure 7E shows that the shortest distance between these stimulation sites and the pLIC parcellation corresponds to M1 cluster of head/face and tongue/larynx, validating the clinical findings obtained intraoperatively.

## 4 Discussion

Here, we present a new potential imaging landmark, within the pLIC, that could serve as a patient-specific refinement tool for targeting of the Vim subnucleus of the thalamus for neurosurgery applications. PICS can be particularly helpful with estimating the electrode (or lesion) location in neurosurgical application by inferring the location of Vim even in standard-of-care images that do not allow direct visualization of thalamic subnuclei. Our findings demonstrate that PICS can not only be identified across several MRI sequences including T1, FGATIR, and T2* (Figure 1) but more clearly and reliably visible using DiMANI in all 15 participants from our sample (Figures 1, 2, 7). Rather than replacing current methods, we suggest the use of PICS as a complementary targeting strategy. Its visibility on routinely acquired data (T1, FGATIR, T2*) provides ease of implementation.

Further, our data show that PICS is visible not only 7 T images, but also on 3 T images (Figure 2), which makes it more scalable for implementation across the majority of DBS centers as 7 T is not yet a routine scanner to be found at the majority of DBS locations. Our findings demonstrate that PICS appears to comprise mostly motor-related fibers (Figures 3–7), bordering laterally the Vim subnucleus, while sometimes partially bordering anterior Vc and posterior Vop (Figures 1, 5 and see (Patriat et al., 2024) for details on how to identify the Vim, Vop, and Vc on DiMANI images). To our knowledge, this is the first work to radiologically parcellate the region of pLIC into functional subregions corresponding to the motor and sensory homunculus in individual patients (Figures 6, 7). Further, our proof-of-concept case demonstrates correspondence between somatotopic organization within the pLIC, based on tractography, and clinical manifestation of tonic contractions in the respective body part, based on intra-operative stimulation testing. These parcellations have the potential to help clinical teams intra- and post-operatively with refining final DBS electrode location or guide potential surgical planning although correlations with clinical outcomes still need to be further studied.

### 4.1 Validity of pLIC parcellations

Our pLIC HMAT parcellation results were similar to prior work using 3T datasets (Archer et al., 2018; Lee and Park, 2022), thus replicating 3 T findings at 7 T at both the group level and the individual level. Furthermore, these results also parallel tracer studies conducted in the non-human primate (Morecraft et al., 2017).

The patient-specific pLIC parcellations at 7T also showed good correspondence to the cross-section of the SMATT atlas with the pLIC. Parcellation of the pLIC using homunculus M1 and S1 tractography has not been extensively explored in humans. Weiss et al. (2015) demonstrated in one intracranial tumor patient, that at the level of pLIC, the M1 foot region was posterior to M1 hand, which itself was posterior to M1 tongue using transcranial magnetic stimulation and dMRI. Further, all three regions were concentrated in the posterior third quarter of the pLIC with maintenance of the anterior–posterior organization, consistent with the location of PICS demonstrated in our results (Figure 6). Our M1 homunculus results are consistent with anatomo-pathological findings (plate 746 from Gray’s anatomy, 1918; King’s College London, 2011) (Gray and Lewis, 1918; Bannister, 2011). Second, using imaging data, Northam et al. (2019) demonstrated that the corticospinal tract (CST), containing fibers responsible for limbs and trunk movements, were located more posteriorly to corticobulbar tracts (CBT), which carry information correlated with head and neck. Finally, non-human primate tracer evidence also suggests that upper limb fibers run posteriorly to orofacial fibers in the pLIC (Morecraft et al., 2017), which is replicated by our results. Although no neuroimaging studies conducting parcellation of the pLIC based on S1 homunculus have been published, to our knowledge, it is believed that the fibers from the S1 homunculus follow a similar pattern to the M1 homunculus in pLIC but located posteriorly to these (Figures 10–22 from Clinical Neuroanatomy) (Waxman, 2013), which also is consistent with our findings (Figure 6).

### 4.2 What is this PICS?

The IC is well known to be a heterogenous white matter structure. In fact, laser microscopy studies have shown that parcellations of the IC can be performed based on the local fiber orientation, density and diameter (Axer and Keyserlingk, 2000). The pLIC can be subdivided into two regions with a small gradient-like transition: one with larger diameter fibers running nearly perpendicular to the ACPC plane, and one with smaller diameter and more transversally oriented fibers (Axer and Keyserlingk, 2000). Axer and Keyserlingk (2000) call them CI 3 and CI 4, respectively. Additionally, they have demonstrated that CI 3 contains the pyramidal tracts (PyT). PyT is comprised of CBT and CST; therefore, given that CBT is more anterior than CST, it remains to be determined whether PICS is the posterior part of CI 3 or the overlapping region between CI 3 and CI 4. Figures 3, 4, 6 show that PICS is made of motor-related fibers. Although Figure 6 shows the majority of CBT tracts anterior to PICS, the inter-subject variability of tractography results for these more ventral ROIs prohibits one from making a definitive statement. This variability is likely due to dMRI/tractography methodology limitations (see below). However, the same figure clearly shows the CST results contained within PICS. These CST tractography results are in line with previous results showing a “hot spot” in a group-level probabilistic map located in a similar location to our PICS (Bürgel et al., 2006). A 7T study reported the presence of a PICS on T2-weighted images in young healthy individuals and attributed this region to CST fibers (Hervé et al., 2011) but they did not show correspondence with tractography. In this study, we unify the tractography results with anatomical observations on structural MRI.

### 4.3 How to visualize PICS?

Figure 1 shows that PICS can be visible on several MRI contrasts (T2*, T1, FGATIR, B0, QSM, DiMANI, Tensor) as a hyperintense or hypointense signal depending on the MRI sequence. dMRI data (B0 and DiMANI) appears to more readily show PICS but the other promising sequences could potentially be optimized to improve the visualization of the landmark further. The visibility of PICS on routinely used sequences such as FGATIR, T1, and T2star suggests a potentially easy translation of the use of PICS in the clinic. Note that we did not see PICS on our T2, but it was visible on the template; while it could be a matter of SNR, it could be the result of comparing images from very different T2 protocols. Further, in Figure 1, while our QSM image appears to show PICS, the template QSM did not. This again may be due to differences with the MRI protocol used in the acquisition of the images that make up the template. Finally, different approaches have been utilized to generate a neuromelanin-sensitive contrast, so while we did not see PICS with the common magnetization transfer-weighted TFL approach, others might be able to view it using a turbo spin echo approach.

### 4.4 Clinical relevance and next steps

After decades of successful experiences with surgical interventions for movement disorders, variability in outcomes can be potentially explained in part by electrode location. Improving targeting accuracy to specific subregions and correlations with outcomes is a growing research field (Vitek et al., 2022; Schrock et al., 2021; Gunalan et al., 2017; Brinda et al., 2023; Ikramuddin et al., 2023). Although recent advances to directly visualize the Vim (Najdenovska et al., 2019; Chiang et al., 2018; Sudhyadhom et al., 2009; Su et al., 2019; Middlebrooks et al., 2021; Patriat et al., 2024) have been proposed, many of these are still lacking routine clinical adoption as visualizing the borders of thalamic subnuclei remains a challenge. Figure 1 shows that the simple PICS landmark could be more easily seen than the thalamic subnuclei on a T1, FGATIR, B0 and T2* image. Therefore, PICS can be potentially used as an extra tool to improve target accuracy even in poor quality cases when thalamic borders cannot be well delineated, as Vim is directly medial to the center of mass of PICS, and that there can be partial overlap between the posterior region of PICS and regions lateral to Vc. Further, the knowledge of PICS corresponding to motor internal capsule allows clinicians to translate it into a clinical marker that can be identified in real time during intraoperative testing and outpatient programming. Given the novelty of PICS, there are currently, to our knowledge, no studies evaluating the clinical validity and applicability of this landmark across different medical conditions, age groups, and MRI protocols and manufacturers. Therefore, future studies could focus on comparing PICS identification across different populations with varying degrees of cortical and subcortical atrophy (either due to age or disorder). This would be an interesting topic to explore further. Subsequent studies could also evaluate potential correlations between lead (or lesion) location relative to PICS and clinical outcomes.”

### 4.5 Limitations

Despite rigorous protocol to ensure quality of the dataset, we acknowledge potential heterogeneity in localizations of the tractography, within the pLIC, from the most ventral cortical ROIs. This is likely due to a suboptimal performance in determining fiber orientation in regions of crossing fibers. Higher b-values and/or multi-shell acquisition schemes should remedy this issue. Additionally, while we did not directly explore the relationship between stimulation-induced side effects and pLIC parcellation, the proof-of-concept presented hopefully exemplifies a hypothesis to serve as a foundation for future exploratory analysis. Future studies should focus on clinically validating PICS as a landmark to help with Vim targeting.

## 5 Conclusion

PICS is a novel imaging landmark located in the pLIC. It appears to be consistently detected in both 3 T and 7 T imaging, and although it is best visible on DiMANI, it can also be detected in other methods. Tractography analysis shows that it is mostly comprised of CST, but not CBT fibers. Given its location lateral to the Vim with the most posterior aspect sometimes located lateral to Vc, it can be potentially used as a tool to further refine surgical targeting, including cases where the quality of preoperative imaging may not provide accurate thalamic subnuclei border detection. Further studies are needed to correlate proximity to PICS and lead/lesion location and clinical outcomes.

## Data Availability

The raw data supporting the conclusions of this article will be made available by the authors, without undue reservation.

## Footnotes

1
https://github.com/VincentStimper/mclahe

2
https://www.lead-dbs.org/

3
www.slicer.org

4
https://dsi-studio.labsolver.org/

5
http://www.nitrc.org/projects/meshmetric3d
